# Exploring Single-Nucleotide Polymorphisms in Primary and Secondary Male Infertility

**DOI:** 10.3390/medsci13030109

**Published:** 2025-08-01

**Authors:** Fatina W. Dahadhah, Mohanad Odeh, Heba A. Ali, Jihad A. M. Alzyoud, Manal Issam Abu Alarjah

**Affiliations:** 1 Department of Basic Dental Sciences, Faculty of Dentistry, The Hashemite University, Zarqa 13115, Jordan; heba_ali@hu.edu.jo (H.A.A.); jihada@hu.edu.jo (J.A.M.A.); 2 Department of Clinical Pharmacy and Pharmacy Practice, Faculty of Pharmaceutical Sciences, The Hashemite University, Zarqa 13115, Jordan; mohanad_odeh@hu.edu.jo; 3 Department of Basic Medical Sciences, Faculty of Medicine, Yarmouk University, Irbid 21193, Jordan; manalabualarjah17@gmail.com

**Keywords:** male infertility, SNPs, mitochondrial genes, primary infertility, secondary infertility

## Abstract

Background/Objectives: Infertility, defined as the failure to achieve pregnancy after one year of regular unprotected intercourse, represents a significant global health challenge, with male factors contributing to approximately 50% of cases. In this epidemiological context, both primary male infertility (the inability to conceive a first child) and secondary male infertility (which occurs when a man who has already fathered a child faces difficulty conceiving again) remain poorly understood at the genetic level. This study explored the role of single-nucleotide polymorphisms (SNPs) in mitochondrial genes (*MT-ND3*, *MT-ND4L*, and *MT-ND4*) in primary and secondary male infertility. Methods: This study analyzed the genotype distributions of SNPs in 68 infertile males (49 with primary infertility and 19 with secondary infertility) using Sanger sequencing. Results: Key findings revealed that studied SNPs were significantly associated with infertility type. Specifically, rs2857285 (T>C,G) in the ND4 gene showed a significant correlation (*p* = 0.023) with the TT genotype, which is prominent in primary infertility. Another SNP, rs28358279 (T>A,C) in the ND4L gene, also demonstrated a significant correlation (*p* = 0.046) with the TT genotype, being more common in primary infertility. In addition, rs869096886 (A>G) in the ND4 gene had a borderline correlation (*p* = 0.051), indicating a possible association between this SNP and reproductive duration. Conclusions: This study emphasizes the potential relevance of mitochondrial malfunction in male infertility, specifically the effects of studied SNPs on sperm survival and function over time. These findings suggest that certain mitochondrial SNPs might be potential biomarkers for infertility risk. Larger studies are needed to confirm these associations and examine the functional effects of these SNPs. Combining genetic analysis with environmental and lifestyle factors could enhance our understanding of male infertility and improve diagnostic and therapeutic strategies.

## 1. Introduction

Infertility is a global health concern affecting approximately 15% of couples worldwide. Male infertility contributes to nearly 50% of these cases, and it is categorized as either primary or secondary infertility [1,2]. Primary infertility is clinically defined as the inability to achieve pregnancy after regular unprotected intercourse for at least one year, without prior successful conception [3]. In contrast, secondary infertility occurs when a man who has previously fathered a child faces difficulty conceiving again [2]. Moreover, other factors may contribute to male infertility, including genetic, hormonal, environmental, and lifestyle factors [4].

Primary male infertility is often linked to severe spermatogenic failure, obstructive azoospermia, or congenital conditions such as Klinefelter syndrome (47,XXY) [5]. Genetic defects are responsible for approximately 15–30% of male infertility cases [1]. In contrast, secondary infertility refers to the condition where parents cannot conceive additional children after a year of attempting, after having had one or more biological children, assuming that the previous births did not involve the use of assisted reproductive technologies or fertility treatments [6]. It can result from acquired conditions, including infections, obesity varicocele, lifestyle changes, exposure to environmental toxins, late-onset genetic defects, or age-related sperm decline [6,7]. Aging, oxidative stress, and DNA fragmentation in sperm are key contributing factors to secondary infertility [8]. Genetic mutations in genes such as *CFTR* (cystic fibrosis transmembrane conductance regulator), AZF (azoospermia factor) regions on the Y chromosome, and NR5A1 (nuclear receptor subfamily 5 group A member 1) have been implicated in both primary and secondary infertility [9].

Mitochondria are recognized as energy-producing organelles within cells [10] and contain a compact circular genome that encodes 13 proteins [11]. In humans, the mitochondrial genome consists of 16,569 base pairs and displays a high mutation rate, which can be attributed to the lack of histones and the presence of limited DNA repair mechanisms [12]. Mutations in mitochondrial DNA (mtDNA) can be associated with various genetic disorders, including some types of male infertility [13]. Many studies have indicated that specific single-nucleotide polymorphisms (SNPs) in mtDNA may be linked to reduced sperm quality and decreased motility, which can impact male fertility [14]. Malfunctioning mitochondria in sperm result in decreased ATP production and increased free radicals, further impacting motility [15,16]. The mitochondrial respiratory chain includes 13 essential proteins across its complexes. Complex I is composed of seven subunits of NADH dehydrogenase, designated *ND1*, *ND2*, *ND3*, *ND4*, *ND4L*, *ND5*, and *ND6*. It is the first enzyme in the electron transport chain and oxidizes the NADH produced during the Krebs cycle. This step is the rate-limiting step in cellular respiration [17]. Significant deletions in mtDNA, including essential genes such as *ATPases 6* and *8*, *ND3*, and *ND4L*, have been noted in asthenozoospermia patients [18,19,20,21]. Moreover, genetic variations in the mitochondrial genes *MT-ND4* and *MT-TL1* were identified only in individuals with asthenospermia, with no such changes observed in the fertile group. These variants are likely to interfere with mitochondrial function and energy metabolism, both of which are critical for supporting healthy sperm motility. The findings indicate a possible link between these gene variations and reduced semen quality, indicating their possible role in the development of male infertility [22]. However, the molecular mechanisms behind male infertility are still not fully understood, and other genetic factors, such as methylenetetrahydrofolate reductase (*MTHFR*) and cystic fibrosis transmembrane conductance regulator (*CFTR*), have also been reported as contributors [23,24].

Genetic variations play crucial roles among other effectors; one type of genetic variation is single-nucleotide polymorphism (SNP) [25], which are variation in a single nucleotide within a DNA sequence that can influence gene expression and protein function, influencing reproductive pathways [26]. Certain SNPs have been associated with male infertility due to their effects on spermatogenesis, sperm motility, and hormonal regulation [27,28].

Mitochondria play a crucial role in spermatogenesis and sperm quality, undergoing significant changes in morphology and function as germ cells develop. Spermatogonia and early spermatocytes contain small, spherical mitochondria with low oxidative phosphorylation (OXPHOS) activity, relying mainly on glycolysis. In later stages, elongated mitochondria perform OXPHOS using lactate and pyruvate from Sertoli cells to meet heightened energy needs. Mature spermatozoa feature a helical sheath of 50–80 mitochondria in the midpiece, generating ATP for motility, where reduced mitochondrial membrane potential is linked to asthenozoospermia [29,30,31,32]. Mitochondria also regulate reactive oxygen species (ROS), which are essential for sperm functions. However, excessive ROS can cause oxidative stress and DNA damage due to the vulnerability of sperm mitochondrial DNA. Furthermore, they regulate germ cell quality through apoptosis and influence steroidogenesis in Leydig cells by initiating testosterone synthesis. Mitochondrial dysfunction from obesity, diabetes, or toxins disrupts ATP production, increases ROS, and can lead to infertility [30,31,32,33,34].

One of the most common examples is SNPs in the follicle-stimulating hormone receptor (*FSHR*) gene, which are correlated with reduced sperm production and hormonal imbalances [35]. Similarly, variations in the *AR* (androgen receptor) gene are associated with impaired testosterone signaling, leading to reduced sperm count and motility [36]. Additionally, SNPs among *MTHFR* (methylenetetrahydrofolate reductase) play a role in increasing oxidative stress, which leads to sperm DNA damage, causing male infertility [37]. Recent research suggests that SNP analysis could be valuable for diagnosing male infertility and developing treatment strategies [38]. Advances in precision medicine and genetic counseling based on SNP profiling may help improve fertility outcomes for affected individuals [39].

The current study aimed to investigate the role of single-nucleotide polymorphisms (SNPs) in the *MTND3*, *MTND4L*, and *MTND4* genes related to both primary and secondary male infertility by comparing their genotypic profiles using Sanger sequencing.

## 2. Materials and Methods

### 2.1. Study Population

Semen samples were collected from subfertile males (aged 26–48 years) attending the In Vitro Fertilization (IVF) Clinic at Royal Medical Services hospital after obtaining Institutional Review Board (IRB) approval. Strict criteria were set for selecting participants, which ruled out individuals older than 50 years, along with those who smoke, consume alcohol, or have experienced chemotherapy or radiotherapy. Additionally, males with varicocele, hormonal imbalances, or chronic illnesses were also excluded from the study. This approach aimed to enable a focused investigation into the causes of subfertility. After applying these criteria a total of 68 participants were enrolled and categorized into two groups: 49 with primary infertility (no prior conception) and 19 with secondary infertility (previous parenthood with current conception difficulties).

### 2.2. Collection and Preparation of Semen Samples

The sperm samples were collected via masturbation after a period of three days of abstinence and placed in sterile containers. The samples were then allowed to liquefy at 37 °C for 30 min. Clinical history and various sperm parameters, including ejaculate volume, count, viscosity, morphology, and motility, were assessed via a Makler counting chamber on the basis of the WHO guidelines [3]. The samples were subjected to discontinuous sperm concentration gradients of 45% and 90% prior to DNA extraction. They were subsequently subjected to centrifugation at 250× *g* for a period of 20 min. The resulting pellet was thoroughly washed, confirmed to be free of other cells microscopically, and stored at −20 °C in preparation for DNA extraction.

### 2.3. Extraction of Mitochondrial DNA

The sperm cells were subjected to incubation on ice with somatic cell lysis buffer (SCLB) for 30 min, followed by two washes with phosphate-buffered saline (PBS), each lasting 10 min. Whole-genome extraction was performed using a QIAamp DNA Mini Kit, after which mitochondrial DNA amplification was conducted via a REPLI-g Mitochondrial DNA Kit (QIAGEN, Hilden, Germany). The quality of the DNA was evaluated using a 260/280 ratio of 1.8 or higher, and the DNA was subsequently stored at −80 °C.

### 2.4. PCR Assay of Mitochondrial Genes

This study focused on mitochondrial genes (*MTND3*, *MTND4L*, and *MTND4*) related to the NADH dehydrogenase CoQ reductase for SNP genotyping. PCR was performed utilizing self-designed primers generated with PRIMER 3 software (Tartu, Estonia). The primers employed in this study were designed using the mitochondrial sequence of humans (accession number NC_012920) from the National Center for Biotechnology Information (NCBI) and subsequently ordered from the Microsynth Seq Lab, Göttingen, Germany (Table S1).

The amplification reaction was conducted in a thirty microliter volume utilizing Thermo Scientific Dream Taq Green PCR Master Mix (2×), adhering to the manufacturer’s guidelines (Thermo Fisher Scientific, Erlangen, Germany). The optimized conditions were applied for the target genes. To verify the success of the PCR amplification, five microliters per sample were analyzed using 1% gel electrophoresis with 1× TBE buffer alongside a 1 kb DNA ladder (New England Biolabs, Ipswich, MA, USA) as a reference, with the gel running at a voltage of 100 volts for 45 min. Subsequently, the gels were stained using a red-safe stain, and the visualization of the DNA was carried out via a UV transilluminator along with Image Lab™ Software (Bio-Rad, Hercules, CA, USA).

### 2.5. MT-DNA Sequencing

The MT-DNA samples were purified and sequenced using the Sanger method at the Microsynth Seq laboratory in Germany. The PCR products were thereafter sequenced with the same PCR primers. Single-nucleotide polymorphisms (SNPs) in *MTND3*, *MTND4L*, and *MTND4* were identified through sequence analysis, referencing human mitochondrial DNA (NC_012920). Subsequently, the sequenced DNA specimens were investigated via FinchTV (Seattle, WA, USA), following the identification of mitochondrial DNA variants via Mutation Surveyor (State College, PA, USA).

### 2.6. Statistical Analysis

Genotype and allele frequencies were compared between the primary infertility group (*N* = 49) and the secondary infertility group (*N* = 19) using the Chi-square test. For 2 × 2 tables, both the Pearson Chi-square and the Likelihood Ratio Chi-square tests were evaluated. When the total sample size exceeded 40 (*N* > 40) and expected cell counts were < 5, the Likelihood Ratio Chi-square was preferred as it provides a more robust and powerful likelihood-based assessment in moderate samples. Fisher’s Exact Test was applied only when any expected cell count was <1, or in cases of small sample size (*N* < 40) where exact inference is necessary. All tests were two-tailed, and a *p*-value ≤ 0.05 was considered statistically significant. Analyses were conducted independently for each SNP without adjustment for multiple comparisons, as each analysis was exploratory [40,41,42,43].

## 3. Results

The age of our participants is reported as a median of 34.0 years (IQR 10.0). Body mass index (BMI) data were not collected in this study. Alcohol consumption and cigarette habits were considered as exclusion criteria; therefore, no participants reported these behaviors (0% prevalence). The frequencies of male infertility diagnoses are as follows: oligoasthenoteratospermia (OAT, 11.76%), asthenoteratospermia (AT, 16.18%), oligoteratospermia (OT, 13.24%), asthenospermia (A, 11.76%), teratospermia (T, 42.65%), and oligospermia (O, 4.41%).

Table S2 presents the genotype distribution of SNPs in *ND4* among 66 infertile individuals, revealing a high frequency of specific genotypes within the patients. Several SNPs show highly skewed distributions, with certain genotypes being nearly fixed. Notably, rs2853495 (GA) shows a 65% prevalence of the mutant-type allele AA over the wild-type allele GG, suggesting strong genetic consistency. However, rs2857284 (T>C) shows a predominance of the TT genotype (71%), whereas rs2853497 (G>A) is characterized by the GG genotype in 92% of individuals. Similarly, rs2853493 (A>G) and rs2853490 (G>A) presented a 97% frequency of a single genotype (AA or GG). Additionally, multiple SNPs, including rs28529320 (T>C), rs2853494 (A>G), rs28609979 (T>C), rs28358286 (C>T), and rs28359168 (A>G), have a 100% prevalence of a single genotype, indicating complete fixation in the population. One of the few SNPs with a more varied distribution was rs869096886 (A>G), where the AA genotype was observed in 76%, while the GG and AG genotypes accounted for 23% and 2%, respectively. The remaining SNPs, rs2857284, rs2853497, and rs28529320, warrant further investigation because of their highly biased distributions and potential role in infertility susceptibility.

As shown in Table 1, 66 infertile individuals were divided into primary (*N* = 47) and secondary (*N* = 19) infertility cases, and Chi-square tests and *p*-values were used to assess their statistical significance. Two SNPs were potentially relevant to infertility type. The first SNP, rs2857285 (T>C,G), stands out with a *p*-value of 0.023, suggesting a potential correlation between primary and secondary infertility cases. For this SNP the CC genotype is predominant (11%) in secondary infertile males compared with 0% in primary infertile males. Another variant, rs869096886 (A>G), has a borderline *p* -value (0.051), indicating a potential trend worthy of further investigation. For this SNP the GG genotype is also more common (42%) in secondary infertile males than in primary infertile males (15%), indicating a potential link between this SNP and reproductive longevity. While most SNPs do not show a significant association with infertility type, rs2857285 (T >C,G) emerges as a potential genetic marker and rs869096886 (A>G) shows a trend that may warrant further investigation.

For the majority of SNPs, such as rs2857284 (T>C), rs2853497 (G>A), and rs3087901 (T >A,C,G), the genotype distributions are similar between primary and secondary infertility cases, with *p*-values exceeding 0.1, suggesting no strong genetic differentiation in those SNPs. Additionally, several SNPs, including rs28529320 (T>C), rs2853494 (A>G), rs28609979 (T>C), and rs28358286 (C>T), show complete fixation of a single genotype across all individuals, making statistical comparison infeasible.

The genotype distribution of SNPs in the *ND4L* gene among 63 infertile males (primary (N = 45) and secondary (N = 18) infertility cases) revealed a strong prevalence of specific genetic variants. Several SNPs appear to be nearly fixed in the population, with limited genetic variability. Notably, rs2854121 (C>T) and rs28532881 (C>A) exhibited a 100% presence of the CC genotype, indicating complete fixation of this allele in the sample. Similarly, rs2853487 (G>A) and rs2853488 (G>A) are dominated by the GG genotype (97%), with only a small proportion carrying alternative alleles. The rs28358280 (A>G) SNP is also highly skewed, with 98% of individuals carrying the AA genotype and only 2% having GG. In rs28358281 (G>A,C) the GG genotype is highly prevalent (90%), whereas the AA and GA genotypes appear at much lower frequencies (6% and 3%, respectively). A similar trend was observed for rs28358279 (T>A, C), where the TT genotype dominated (94%), with the CC genotype appearing in only 6% of individuals. There was a high degree of genetic uniformity within the sample, with several SNPs showing little to no variation. Both the rs2854121 and rs28532881 SNPs presented 100% CC genotype prevalence. The limited presence of alternative alleles in other SNPs, such as rs28358280 and rs28358281, further highlights potential genetic predispositions that warrant further investigation (Table S3).

While most SNPs did not significantly differ between the two groups, rs28358279 (T>A,C) exhibited a notable *p*-value of 0.046, suggesting a possible association between primary and secondary infertility cases. For this SNP, the CC genotype is predominant, with a slightly greater occurrence in secondary infertility (17%) than in primary infertility (2%). In comparison, the TT genotype is highly prevalent (98%) in primary infertility cases. For other SNPs, such as rs28358280 (A>G) and rs28358281 (G>A,C), the distributions are highly skewed toward a dominant genotype (AA and GG, respectively), although their *p*-values (0.111 and 0.474, respectively) do not indicate strong statistical significance between the two groups. Similarly, rs2853487 (G>A) and rs2853488 (G>A) are dominated by the GG genotype (97%), with the AA or GA genotypes appearing in only 3% of cases, and both SNPs show no significant associations (*p* = 0.495). Additionally, rs2854121 (C>T) and rs28532881 (C>A) are completely fixed in the population, with 100% of individuals carrying the CC genotype, making statistical comparisons infeasible (Table 2).

The genotype distribution of SNPs in *ND3* among 68 infertile individuals highlights the strong predominance of specific genotypes. Several SNPs exhibited highly skewed distributions, suggesting limited genetic variability within the two groups. For rs2853826 (A>G,T) the AA genotype was the most common (54%), followed by the GG genotype (44%), whereas the AG genotype appeared in only 1% of individuals. Similarly, in rs28435660 (G>A) the GG genotype dominated (90%), with GA and AA occurring at much lower frequencies (6% and 4%, respectively). Certain SNPs, including rs28358275 (T>C), rs28358278 (C>T), and rs41467651 (G>A), show an overwhelming presence of a single genotype, with the TT or GG genotype occurring in more than 90% of cases. Additionally, rs3899188 (T>C) and rs28673954 (T>C) exhibit an almost complete fixation of the TT genotype (99%), whereas their alternative alleles are present in only 1% of cases. These findings suggest that certain *ND3* SNPs are highly conserved within this population, with a few dominant genotypes occurring at very high frequencies. The near-fixation of the TT and GG genotypes in several SNPs indicates potential genetic markers that may be relevant to further research on population genetics and disease susceptibility (Table S4).

While all SNPs in Table 3 show no significant differences between primary and secondary infertility cases, a few variants exhibit notable trends in genotype distribution. For rs2853826 (A>G,T) the AA genotype was the most common (54%), followed by the GG genotype (44%), whereas the AG genotype was rare (2%). However, the *p*-value (0.270) did not indicate a significant association between the two infertility groups. Similarly, rs28435660 (G>A) is dominated by the GG genotype (90%), with AA and GA occurring in only 4% and 6% of individuals, respectively (*p* = 0.151). For rs28358275 (T>C) and rs28358278 (C>T) the TT genotype is highly predominant (91% and 96%, respectively), with very few individuals carrying alternative genotypes (*p* = 0.451 and 0.270, respectively). Additionally, rs3899188 (T>C) and rs28673954 (T>C) show near-complete fixation of the TT genotype (99%), whereas alternative alleles appear in only 1% of cases (*p* = 0.530 and 0.106, respectively). While no SNPs reached statistical significance, the high prevalence of specific genotypes suggests that studied SNPs in *ND3* may be genetically conserved within this population.

For the allele frequencies test most SNPs did not show statistical relevance, implying a limited role in differentiating types of infertility. In ND4, however, rs2857285 revealed a notable correlation between primary and secondary male infertility (OR = 24.65, 95% CI: 1.29–469.92, *p* = 0.03), and rs69096886 had an OR of 3.83 (*p* = 0.002). Similarly, in ND4L rs28358279 demonstrated a strong association between the two groups (OR = 8.80, 95% CI: 1.68–45.96, *p* = 0.001), whereas other SNPs (rs28358280, rs28358281, rs2853487, and rs2853488) presented elevated but non-significant ORs (2.54–13.12), presumably attributable to restricted sample sizes. Among the ND3 polymorphisms, rs193309227 (OR = 2.82, *p* = 0.12) and rs28673954 (OR = 7.88, *p* = 0.21) presented suggestive trends but lacked statistical power. These results generally identify rs2857285 and rs2858279 as possible risk markers, but they also emphasize the need for larger studies to clarify the genetic causes of infertility (Tables S5–S7).

## 4. Discussion

This study analyzed the frequency of single-nucleotide polymorphisms (SNPs) in mitochondrial genes (*MT-ND4*, *MT-ND4L*, and *MT-ND3*) and their potential correlation with primary and secondary male infertility. The genotype distributions for several SNPs were analyzed among 68 infertile individuals, with 49 classified as having primary infertility and 19 as having secondary infertility. These findings provide insights into whether genetic variations in mitochondrial DNA contribute to infertility type and whether studied SNPs may serve as potential biomarkers for reproductive dysfunction.

A comparative analysis of genotype distributions indicated that specific SNPs showed significant trends that could have clinical importance between the primary and secondary infertility groups. However, minimal statistically significant differences were observed in other SNPs, suggesting substantial genetic similarity between the two groups.

In the *ND4* gene, rs2857285 (T>C,G) was significantly associated with infertility type (*p* = 0.023). The TT genotype was found in 100% of primary infertility cases and in 89% of secondary infertility cases, with the CC genotype exclusively present in secondary infertility cases (11%). These findings suggest that this variant may play a role in distinguishing primary infertility from secondary infertility. The result of this missense polymorphism is the conversion of cysteine (major allele) into tryptophan (minor allele) at position 52; this might significantly affect the structural consequences of *ND4*, mainly in the protein’s folding and stability or enzymatic interactions within mitochondrial complex I because these amino acids have different chemical properties, thereby impairing oxidative phosphorylation. Tryptophan is a large, hydrophobic amino acid with an aromatic side chain; it might generate steric strain or impact the enzyme’s function due to its bulkiness and hydrophobicityIn contrast, cysteine is polar and smaller in size, which might alleviate this strain. However, it could also impact the enzyme’s performance differently because of its crucial role in protein structure through disulfide bridge formation, which helps stabilize protein folding [44]. This is consistent with studies demonstrating that cysteine substitutions (e.g., C→A) can disrupt disulfide-dependent structural integrity and abolish bioactivity in diverse proteins, including antimicrobial peptides [45]. Such structural disruptions could lower ATP synthesis, which is critical for sperm motility, and exacerbate oxidative stress, leading to infertility [46]. Two researchers investigated relationships between this SNP and other diseases, including ovarian cancer (OvCa) and peripheral artery disease (PAD). They found a nominally significant association; nevertheless, these results did not remain significant after adjustments for multiple tests. Notably, the minor allele frequency (MAF) of rs2857285 was very low, and the study was underpowered to assess rare variants robustly. Moreover, the haplogroups were not significantly associated with OvCa risk or PAD [47,48].

Another SNP, rs869096886 (A>G), had a borderline *p*-value of 0.051, with the AA genotype being more frequent in primary infertility cases (83%) than in secondary infertility cases (58%). In comparison, the GG genotype was more prevalent in patients with secondary infertility (42%). This difference could indicate a potential link between this SNP and reproductive longevity. This variant could impair the efficiency of Complex I in the mitochondrial respiratory chain. This can eventually lead to a reduction in ATP production, which is critical for sperm motility, and potentially cause an increase in oxidative stress and damage to sperm DNA, thus reducing fertility parameters, particularly in cases where sperm motility is compromised such as asthenozoospermia [49].

In the *ND4L* gene, rs28358279 (T>A,C) was significantly associated with infertility type (*p*= 0.046). The TT genotype was highly prevalent in primary infertility cases (98%), but was slightly lower in secondary infertility cases (83%) where the CC genotype appeared at a higher frequency (17%). This SNP occurs in the *ND4L* gene and may lead to changes in mitochondrial functions by disrupting the efficiency of Complex I in the electron transport chain and ATP production. The SNP has been associated with male infertility, especially asthenozoospermia where compromised sperm motility is a major factor [50]. These results demonstrated that although the rs28358279 SNP is a synonymous polymorphism, it can nevertheless contribute to the development and progression of male infertility. A recent study revealed that rs28358279 is associated with cardiovascular traits [51].

In the *ND3* gene of the mitochondrial genome, while no SNPs reached statistical significance some exhibited notable genotype frequency differences. The missense variant rs2853826 results from A > G at position 10398 and changes the amino acid from threonine to alanine (Thr114Ala). This SNP is one of the most extensively studied mitochondrial polymorphisms; for this SNP the AA genotype was slightly more common in primary infertility cases (55%) than in secondary infertility cases (53%), whereas the GG genotype occurred at nearly the same frequency (45% vs. 42%, respectively). Notably, there is a link to reactive oxygen species (ROS) in mitochondria, which results in oxidative stress and damage to mitochondrial DNA (mtDNA). Therefore, it could correlate with mitochondrial dysfunction in sperm, affecting their ability to move toward and fertilize an egg, thus impairing motility [52].

The role of these SNPs in male infertility is likely linked to their impact on mitochondrial function, which is essential for sperm motility and energy production. Previous studies have demonstrated that mitochondrial gene mutations can lead to impaired oxidative phosphorylation, increased reactive oxygen species (ROS) production, and reduced ATP levels, all of which negatively affect sperm function [38].

These findings suggest that specific genetic variants may influence infertility susceptibility (rs2857285, *p* = 0.023; rs28358279, *p* = 0.046; and rs869096886, *p* = 0.051), although larger studies are needed to confirm these associations.

## 5. Limitations

The role of mitochondrial DNA polymorphisms in male infertility is not fully understood, especially between primary and secondary cases. This analytical approach, in which two well-defined patient groups are compared, aligns with established methodologies used in other genetic studies of reproductive disorders [53,54]. The dataset serves as a reference for future research by providing baseline frequency data for mitochondrial variants, identifying specific polymorphisms related to sperm function and energy metabolism, and establishing protocols for mitochondrial genome analysis in male infertility. While larger cohorts are necessary for more definitive confirmation, these findings provide an essential foundation for understanding the role of mitochondrial genetic variation in infertility. Future studies should include broader patient populations and incorporate normozoospermic controls, which will help to better differentiate between general infertility markers and those specific to primary or secondary cases.

## 6. Conclusions

This study identified a few SNPs with potential relevance to primary and secondary male infertility, particularly rs2857285 (T>C,G), rs28358279 (T>A,C), and rs869096886 (A>G). While most SNPs did not significantly differ between infertility types, the presence of specific genetic variations suggests the potential role of mitochondrial dysfunction in sperm viability over time. These findings contribute to the growing body of research on the genetic factors underlying male infertility and highlight the need for further studies with larger sample sizes to confirm these associations. Future research should explore the functional effects of these SNPs on mitochondrial activity and assess their potential as biomarkers for infertility risk. Additionally, integrating genetic analysis with environmental and lifestyle factors may provide a more comprehensive understanding of male infertility and improve the accuracy of diagnostic and therapeutic approaches.

## Figures and Tables

**Table 1:** Genotype frequencies of *MTND4* polymorphisms between primary and secondary infertility.

SNPs (ND4)	Genotype	Infertility		Total	*p* Value
		Primary	Secondary		
rs2853495	AA	29 (62%)	14 (74%)	43 (65%)	0.355
	GG	18 (38%)	5 (26%)	23 (35%)	
rs2857284	CC	13 (28%)	4 (21%)	17 (26%)	0.708
	TC	1 (2%)	1 (5%)	2 (3%)	
	TT	33 (70%)	14 (74%)	47 (71%)	
rs2853496	AA	5 (11%)	3 (16%)	8 (12%)	0.260
	AC	0 (0%)	1 (5%)	1 (2%)	
	GA	3 (6%)	0 (0%)	3 (4%)	
	GG	39 (81%)	15 (79%)	54 (82%)	
rs2853497	AA	1 (2%)	1 (5%)	2 (3%)	0.255
	GA	1 (2%)	2 (11%)	3 (5%)	
	GG	45 (96%)	16 (84%)	61 (92%)	
rs3087901	CC	4 (9%)	1 (5%)	5 (8%)	0.652
	TT	43 (91%)	18 (95%)	61 (92%)	
rs2853493	AA	45 (96%)	19 (100%)	64 (97%)	0.361
	GG	2 (4%)	0 (0%)	2 (3%)	
rs2853490	AA	1 (2%)	1 (5%)	2 (3%)	0.501
	GG	46 (98%)	18 (95%)	64 (97%)	
rs3088053	AA	45 (96%)	17 (89%)	62 (94%)	0.334
	GG	2 (4%)	2 (11%)	4 (6%)	
rs2853491	CC	46 (98%)	18 (95%)	64 (97%)	0.501
	TT	1 (2%)	1 (5%)	2 (3%)	
rs2857285	CC	0 (0%)	2 (11%)	2 (3%)	0.023
	TT	47 (100%)	17 (89%)	64 (97%)	
rs28358282	TC	0 (0%)	1 (5%)	1 (2%)	0.113
	TT	47 (100%)	18 (95%)	65 (98%)	
rs28594904	AA	1 (2%)	0 (0%)	1 (2%)	0.522
	GG	46 (98%)	19 (100%)	65 (98%)	
rs28669780	AA	1 (2%)	0 (0%)	1 (2%)	0.522
	CC	46 (98%)	19 (100%)	65 (98%)	
rs28415973	CC	0 (0%)	1 (5%)	1 (2%)	0.113
	TT	47 (100%)	18 (95%)	65 (98%)	
rs28471078	CC	1 (2%)	0 (0%)	1 (2%)	0.522
	TT	46 (98%)	19 (100%)	65 (98%)	
rs55714831	CC	46 (98%)	19 (100%)	65 (98%)	0.522
	CT	1 (2%)	0 (0%)	1 (2%)	
rs28358283	AA	46 (98%)	19 (100%)	65 (98%)	0.522
	GG	1 (2%)	0 (0%)	1 (2%)	
rs75214962	CC	47 (100%)	18 (95%)	65 (98%)	0.113
	TT	0 (0%)	1 (5%)	1 (2%)	
rs28529320	TT	47 (100%)	19 (100%)	66 (100%)	NA
rs2853494	AA	47 (100%)	19 (100%)	66 (100%)	NA
rs28609979	TT	47 (100%)	19 (100%)	66 (100%)	NA
28358286	CC	47 (100%)	19 (100%)	66 (100%)	NA
rs28359168	AA	47 (100%)	19 (100%)	66 (100%)	NA
28384199	CC	46 (98%)	19 (100%)	65 (98%)	0.522
	GG	1 (2%)	0 (0%)	1 (2%)	
rs3915952 Merged to rs869096886	AA	39 (83%)	11 (58%)	50 (75%)	0.051
	AG	1 (2%)	0 (0%)	1 (2%)	
	GG	7 (15%)	8 (42%)	15 (23%)	

NA: Not applicable.

**Table 2:** Genotype frequencies of *MTND4L* polymorphisms between primary and secondary infertility.

SNP (ND4L)	Genotype	Infertility		Total	*p* Value
		Primary	Secondary		
rs28358280	AA	45 (100%)	17 (94%)	62 (98%)	0.111
	GG	0 (0%)	1 (6%)	1 (2%)	
rs28358281	AA	2 (4%)	2 (11%)	4 (6%)	0.474
	GA	1 (2%)	1 (6%)	2 (3%)	
	GG	42 (94%)	15 (83%)	57 (90%)	
rs28358279	CC	1 (2%)	3 (17%)	4 (6%)	0.046
	TT	44 (98%)	15 (83%)	59 (94%)	
rs2853487	AA	1 (2%)	1 (6%)	2 (3%)	0.495
	GG	44 (98%)	17 (94%)	61 (97%)	
rs2853488	GA	1 (2%)	1 (6%)	2 (3%)	0.495
	GG	44 (98%)	17 (94%)	61 (97%)	
rs2854121 (has merged into rs193302933)	CC	45 (100%)	18 (100%)	63 (100%)	NA
rs28532881	CC	45 (100%)	18 (100%)	63 (100%)	NA

NA: Not applicable.

**Table 3:** Genotype frequencies of *MTND3* polymorphisms between primary and secondary infertility.

SNPs (ND3)	Genotype	Infertility		Total	*p* Value
		Primary	Secondary		
rs2853826	AA	27 (55%)	10 (53%)	37 (54%)	0.270
	AG	0 (0%)	1 (5%)	1 (2%)	
	GG	22 (45%)	8 (42%)	30 (44%)	
rs28435660	AA	1 (2%)	2 (11%)	3 (4%)	0.151
	GA	4 (8%)	0 (0%)	4 (6%)	
	GG	44 (90%)	17 (89%)	61 (90%)	
rs28358275 (has merged into rs193302927)	CC	2 (4%)	2 (11%)	4 (6%)	0.451
	TC	1 (2%)	1 (5%)	2 (3%)	
	TT	46 (94%)	16 (84%)	62 (91%)	
rs28358278	CC	46 (94%)	19 (100%)	65 (96%)	0.270
	TT	3 (6%)	0 (0%)	3 (4%)	
rs41467651	AA	2 (4%)	0 (0%)	2 (3%)	0.187
	GA	0 (0%)	1 (5%)	1 (1%)	
	GG	47 (96%)	18 (95%)	65 (96%)	
rs3899188	CC	1 (2%)	0 (0%)	1 (1%)	0.530
	TT	48 (98%)	19 (100%)	67 (99%)	
rs28358277	AA	1 (2%)	0 (0%)	1 (1%)	0.225
	GA	0 (0%)	1 (5%)	1 (1%)	
	GG	48 (98%)	18 (95%)	66 (98%)	
rs28673954	TC	0 (0%)	1 (5%)	1 (1%)	0.106
	TT	49 (100%)	18 (95%)	67 (99%)	

## Data Availability

Data are available on request owing to privacy/ethical restrictions.

## Supplementary Materials

The following supporting information can be downloaded at: https://www.mdpi.com/article/10.3390/medsci13030109/s1, Table S1: Primers used for PCR amplification of *Mt-Nd3*, *Mt-Nd4L*, *Mt-Nd4* genes.; Table S2: Genotype frequencies of MTND4 polymorphisms; Table S3: Genotype frequencies of *MTND4L* polymorphisms; Table S4: Genotype frequencies of *MTND3* polymorphisms; Table S5: Allele frequencies of *MTND4* polymorphisms between primary and secondary infertility; Table S6: Allele frequencies of *MTND4L* polymorphisms between primary and secondary infertility; Table S7: Allele frequencies of *MTND3* polymorphisms between primary and secondary infertility.

## Abbreviations

The following abbreviations are used in this manuscript:

Ala	Alanine
Arg	Arginine
Asn	Asparagine
ATP	Adenosine triphosphate
AZF	Azoospermia Factor Region
Bp	Base pair
CFTR	Cystic fibrosis transmembrane conductance regulator
Cys	Cysteine
DNA	Deoxyribonucleic Acid
F	Forward primer
Gln	Glutamine
Glu	Glutamic acid
Gly	Glycine
I	Internal primer
Ile	Isoleucine
Leu	Leucine
Lys	Lysine
Met	Methionine
mtDNA	Mitochondrial Deoxyribonucleic Acid
Mt-ND3	Mitochondrial NADH dehydrogenase subunit 3
Mt-ND4L	Mitochondrial NADH dehydrogenase subunit 4 L
Mt-ND4	Mitochondrial NADH dehydrogenase subunit 4
NADH	Nicotinamide adenine dinucleotide hydride
NR5A1	Nuclear receptor subfamily 5 group A member 1
PCR	Polymerase chain reaction
Pro	Proline
R	Reverse primer
ROS	Reactive oxygen species
SCLB	Somatic cell lysis buffer
Ser	Serine
SNP	Single nucleotide polymorphism
TBE	Tris-borate-EDTA
Thr	Threonine
Trp	Tryptophan
Tyr	Tyrosine
UV	Ultraviolet
V	Volt
Val	Valine
WHO	World Health Organization
