# Supplementary material for: Exploring Single-Nucleotide Polymorphisms in Primary and Secondary Male Infertility
Source: Med Sci (Basel). 2025 Aug 1;13(3):109. doi: 10.3390/medsci13030109 (PMC12371913; doi:10.3390/medsci13030109)
Supplement: Supplementary file 1 [file medsci-13-00109-s001.zip › medsci-3737859-supplementary.pdf]

Supplementary Material

Table S1: Primers used for PCR amplification of *Mt-Nd3*, *Mt-Nd4L*, *Mt-Nd4* genes.

Primers	Sequences (5′ → 3′)	The length of amplified product (bp)
**Nd3.F**	CCAATTAACTAGTTTTG	420 bp
**Nd3.R**	GAGTCGAAATCATTCGT	
**Nd4L.F**	GATTTCGACTCATTAAATT	376 bp
**Nd4L.R**	CATGTCAGTGGTAGTAATAT	
**Nd4.F**	CTACGTACATAACCTAAACC	1432 bp
**Nd4.R**	CTGATGTTTTGGTTAAAC	

Table S2. Genotype frequencies of *MTND4* polymorphisms

SNPs (ND4)	Nucleotide change	Codon change	Amino acid change	Genotype	Total (n=66)	Frequency
rs2853495	G>A	[GGG]> [GGA]	Gly320Gly	AA	43	65%
				GG	23	35%
rs2857284	T>C	[CCT]> [CCC]	Pro38Pro	CC	17	26%
				TC	2	3%
				TT	47	71%
rs2853496	G>A,C	[ACG]> [ACA]	Thr385Thr	AA	8	12%
				AC	1	2%
				GA	3	5%
				GG	54	82%
rs2853497	G>A	[TGG]> [TGA]	Trp416Trp	AA	2	3%
				GA	3	5%
				GG	61	92%
rs3087901	T>A,C,G	[CTT]> [CTC]	Leu395Leu	CC	5	8%
				TT	61	92%
rs2853493	A>G	[TTA] > [TTG]	Leu236Leu	AA	64	97%
				GG	2	3%
rs2853490	G>A	[CAG]> [CAA]	Gln139Gln	AA	2	3%
				GG	64	97%
rs3088053	A>C,G	[CTA] >[CTG]	Leu351Leu	AA	62	94%
				GG	4	6%
rs2853491	C>T	[AAC]> [AAT]	Asn192Asn	CC	64	97%
				TT	2	3%
rs2857285	T>C,G	[TGT] > [TGC]	Cys52Trp	CC	2	3%
				TT	64	97%
rs28358282	T>C	[CTT] > [CTC]	Leu17Leu	TC	1	2%
				TT	65	98%
rs28594904	G>A,C	[AGT] > [AAT]	Ser86Asn	AA	1	2%

Table S3. Genotype frequencies of *MTND4L* polymorphisms

SNPs (ND4L)	Nucleotide change	Codon change	Amino acid change	Genotype	Total (n=63)	Frequency
rs28358280	A>G	[ATA]>[ATG]	Met27 Met	AA	62	98%
				GG	1	2%
rs28358281	G>A,C	[TCG]>[TCA]	Ser39Ser	AA	4	6%
				GA	2	3%
				GG	57	90%
rs28358279	T>A,C	N/A	N/A	CC	4	6%
				TT	59	94%
rs2853487	G>A	[CTG]>[CTA]	Leu40Leu	AA	2	3%
				GG	61	97%
rs2853488	G>A	[GTG]>[GTA]	Val73Val	GA	2	3%
				GG	61	97%
rs2854121	C>T	[GTC]>[GTT]	Val65Val	CC	63	100%
rs28532881	C>A	[TGC]>[TGA]	Cys98Trp	CC	63	100%

Table S4. Genotype frequencies of *MTND3* polymorphisms.

SNPs (ND3)	Nucleotide change	Codon change	Amino acid change	Genotype	Total (n=68)	Frequency
rs2853826	A>G,T	[ACC]>[GCC]	Thr114Ala	AA	37	54%
				AG	1	1%
				GG	30	44%
rs28435660	G>A	[GCC]>[ACC]	Ala99Thr	AA	3	4%
				GA	4	6%
				GG	61	90%
rs28358275	T>C	[ATT]>[ATC]	Ile60Ile	CC	4	6%
				TC	2	3%
				TT	62	91%
rs28358278	C>T	[ACC]>[ACT]	Thr114Thr	CC	65	96%
				TT	3	4%
rs41467651	G>A	[CTG]>[CTA]	Leu84Leu	AA	2	3%
				GA	1	1%
				GG	65	96%
rs3899188	T>C	[ATT]>[ATC]	Ile19Ile	CC	1	1%
				TT	67	99%
rs28358277	G>A	[GAG]>[GAA]	Glu105Glu	AA	1	1%
				GA	1	1%
				GG	66	97%
rs28673954	T>C	[TAT]>[TAC]	Tyr104Tyr	TC	1	1%
				TT	67	99%

Table S5. Allele frequencies of *MTND4* polymorphisms between primary and secondary infertility.

SNP (ND4)	Allele	Primary infertility (N)	Secondary infertility (N)	OR	95% CI		*P* value
					Lower	Upper	
rs2853495	G	36	10	1.70	0.76	4.00	0.1936
	A	58	28				
rs2857284	T	67	29	0.77	0.3223	1.84	0.56
	C	27	9				
rs2853496	G	81	30	1.45	0.5296	3.99	0.47
	A	13	7				
rs2853497	G	91	34	3.57	0.7590	16.78	0.11
	A	3	4				
rs3087901	T	86	36	0.60	0.12	2.95	0.53
	C	8	2				
rs2853493	A	90	38	0.26	0.01	4.97	0.37
	G	4	0				
rs2853490	G	92	36	2.56	0.35	18.84	0.36
	A	2	2				
rs3088053	A	90	34	2.65	0.63	11.18	0.19
	G	4	4				
rs2853491	C	92	36	2.56	0.35	18.84	0.36
	T	2	2				
rs2857285	T	94	34	24.65	1.29	469.92	**0.03***
	C	0	4				
rs28358282	T	94	36	12.95	0.6068	276.19	0.10
	C	0	2				
rs28594904	G	92	38	0.48	0.02	10.24	0.64
	A	2	0				

rs28669780	C	92	38	0.48	0.02	10.24	0.64
	A	2	0				
rs28415973	T	94	36	12.95	0.6068	276.19	0.10
	C	0	2				
rs28471078	T	92	38	0.48	0.02	10.24	0.64
	C	2	0				
rs55714831	C	93	38	0.81	0.0323	20.31	0.90
	T	1	0				
rs28358283	A	92	38	0.48	0.02	10.24	0.64
	G	2	0				
rs75214962	C	94	36	12.95	0.6068	276.19	0.10
	T	0	2				
28384199	C	92	38	0.48	0.02	10.24	0.64
	G	2	0				
rs3915952 Merged to rs869096886	A	79	22	3.83	1.64	8.95	**0.002***
	G	15	16				

Table S6. Allele frequencies of *MTND4L* polymorphisms between primary and secondary infertility.

SNP (ND4L)	Allele	Primary infertility (N)	Secondary infertility (N)	OR	95% CI		*P* value
					Lower	Upper	
rs28358280	A	90	34	13.12	0.61	280.19	0.10
	G	0	2				
rs28358281	G	85	31	2.74	0.7428	10.12	0.13
	A	5	5				
rs28358279	T	88	30	8.80	1.68	45.96	**0.001***
	C	2	6				
rs2853487	G	88	34	2.59	0.35	19.12	0.35
	A	2	2				
rs2853488	G	89	35	2.54	0.15	41.79	0.51
	A	1	1				

Table S7. Allele frequencies of *MTND3* polymorphisms between primary and secondary infertility.

SNPs (ND3)	Allele	Primary infertility (N)	Secondary infertility (N)	OR	95% CI		P value
					Lower	Upper	
rs2853826	A	54	21	0.99	0.47	2.11	0.99
	G	44	17				
rs28435660	G	92	34	1.80	0.48	6.79	0.38
	A	6	4				
rs28358275 (has merged into rs193302927)	T	93	33	2.82	0.77	10.36	0.12
	C	5	5				
rs28358278	C	92	38	0.18	0.01	3.36	0.25
	T	6	0				
rs41467651	G	94	37	0.64	0.07	5.87	0.69
	A	4	1				
rs3899188	T	96	38	0.50	0.02	10.68	0.66
	C	2	0				
rs28358277	G	96	37	1.30	0.11	14.74	0.83
	A	2	1				
rs28673954	T	98	37	7.88	0.31	197.75	0.21
	C	0	1				
